# Testing different models of pharmacy-based HIV pre- and post-exposure prophylaxis initiation and management in Kenya: protocol for a cluster-randomized controlled trial

**DOI:** 10.21203/rs.3.rs-5968938/v1

**Published:** 2025-03-27

**Authors:** Tabitha Kareithi, Stephanie D. Roche, Victor Omollo, Patricia A. Ong’wen, Catherine Kiptinness, Peris Otieno, Lawrence Juma, Rachel C. Malen, Kendall Harkey, Micah O. Anyona, Kelly Curran, Preetika Banerjee, Eunice Gichuru, Magdaline Asewe, Kaiyue Yu, Jillian Pintye, Melissa Mugambi, Parth D. Shah, Monisha Sharma, Allison Meisner, Daniel Were, Kenneth Ngure, Elizabeth A. Bukusi, Katrina F. Ortblad

**Affiliations:** Kenya Medical Research Institute; Fred Hutch Cancer Center: Fred Hutchinson Cancer Center; Kenya Medical Research Institute; Jhpiego Corporation; Kenya Medical Research Institute; Kenya Medical Research Institute; Kenya Medical Research Institute; Fred Hutch Cancer Center: Fred Hutchinson Cancer Center; Fred Hutch Cancer Center: Fred Hutchinson Cancer Center; Jhpiego Corporation; Jhpiego Corporation; Fred Hutch Cancer Center: Fred Hutchinson Cancer Center; Kenya Medical Research Institute; Kenya Medical Research Institute; Fred Hutch Cancer Center: Fred Hutchinson Cancer Center; University of Washington; University of Washington; Fred Hutch Cancer Center: Fred Hutchinson Cancer Center; University of Washington; Fred Hutch Cancer Center: Fred Hutchinson Cancer Center; Jhpiego Corporation; Kenya Medical Research Institute; Kenya Medical Research Institute; Fred Hutch Cancer Center: Fred Hutchinson Cancer Center

**Keywords:** pharmacy, pre-exposure prophylaxis (PrEP), post-exposure prophylaxis (PEP), HIV prevention, differentiated service delivery (DSD), implementation science, Kenya

## Abstract

**Background:**

In Kenya, as in many African countries, private pharmacies are ubiquitous, frequently accessed, and underutilized for the delivery of HIV prevention services. Whether enabling private pharmacies to initiate and manage clients on HIV pre- and post-exposure prophylaxis (PrEP and PEP) leads to greater uptake and continuation than the current standard–pharmacy referral to clinic-based PrEP/PEP–is unknown. To address this gap and inform how private pharmacies might partner with the public sector, we are testing several models of pharmacy-delivered PrEP/PEP in comparison to the current standard.

**Methods:**

The Pharm PrEP cRCT is a 60-pharmacy, four-arm cluster-randomized controlled trial ongoing in Central and Western Kenya (first enrollment: 26 June 2023). Eligible pharmacies were licensed by the government, had a private room, and were willing to complete research activities (including a three-day provider training). Study pharmacies were randomized 1:1:1:1 to: 1) *client-sustained delivery*, in which clients pay pharmacies 250 KES (~$2 USD) per PrEP/PEP visit, 2) *implementor-sustained delivery*, in which clients pay nothing and implementors pay pharmacies 250 KES per PrEP/PEP visit, 3) *implementor-sustained + counselor-supported delivery*, in which clients pay nothing, delivery is supported by an HIV testing services (HTS) counselor, and implementors pay pharmacies 100 KES (~$1 USD) per PrEP/PEP visit, or 4) *referral (control)*, in which clients pay nothing and implementors pay pharmacies 100 KES per referral to clinic-based PrEP/PEP. Pharmacies delivering PrEP/PEP receive supporting commodities free from government stock. Primary outcomes are PrEP initiation and continuation (any refilling) reported by clients 60 days post-enrollment; secondary outcomes include PEP initiation, PEP-to-PrEP transition, repeat PEP use, PrEP/PEP initiation, and PrEP/PEP continuation at 60 and 270 days post-enrollment. Primary analyses will compare each intervention arm to the control; secondary analyses will compare intervention arms to one another. We will additionally assess implementation outcomes (e.g., acceptability, feasibility, cost) from client and provider perspectives.

**Discussion:**

This trial will generate evidence on the potential benefits of leveraging private pharmacies for delivery of PrEP and PEP and the relative effectiveness of pharmacy delivery when subsidized by clients, implementors, and/or supported by HTS counselors. The findings may inform enabling policy and approaches for scale-up.

**Trial registration:**

ClinicalTrials.gov: NCT05842122

## BACKGROUND

In 2024, there were 450,000 new HIV infections in Eastern and Southern Africa,^1^ 34% of all global infections; to help curb the HIV epidemic, new models of delivery for highly-effective biomedical HIV pre- and post-exposure prophylaxis (PrEP and PEP) are needed.^2–4^ In many African countries, free oral PrEP and PEP services are primarily delivered at public healthcare clinics, where client barriers to access–including long travel distances and wait times, lack of privacy, and stigma–continue to challenge PrEP/PEP uptake and continuation.^5, 6^ Additionally, despite guideline revisions recommending PEP use for all individuals with recent HIV exposure^7^ in many African countries, PEP continues to be offered primarily to individuals reporting occupational HIV exposure or sexual assault;^8, 9^ provider bias and low public awareness have further limited PEP use.^10, 11^ To reach additional people who could benefit from biomedical HIV prevention interventions, new models of PrEP/PEP delivery are needed outside clinic settings.

Private, community pharmacies–ubiquitous in many African countries and a common first stop for health services^12–14^–can be leveraged to deliver HIV prevention services. In formative qualitative research led by our team in Kenya, many pharmacy clients said they prefer accessing health services at private pharmacies over traditional health clinics, citing better convenience, privacy, and quality of care.^12–14^ Pilot studies in Kenya led by our team demonstrated the feasibility of PrEP/PEP delivery by pharmacy providers, when guided by a standardized prescribing checklist to identify clients unlikely to have any drug contraindications and overseen by a remote clinician.^6, 15, 16^ Findings from these pilots indicate that PrEP/PEP are in demand at pharmacies and that pharmacies are capable not only of reaching clients underrepresented in clinic-based PrEP/PEP programs (e.g., unmarried individuals, men), but also of achieving levels of PrEP continuation on par with those observed at public clinics.^15, 16^ Key challenges to the scale-up of this model, however, include policy gaps (e.g., limits to pharmacy providers’ scope of practice, which currently excludes delivery of HIV testing and antiretroviral treatment [ART]); ambiguity around how delivery would be financed; and known barriers to implementation feasibility, such as PrEP/PEP delivery time often exceeding what pharmacy providers have available.

To generate evidence to inform whether and how Kenya implements PrEP/PEP services at private pharmacies, we are conducting a superiority, cluster-randomized controlled trial (cRCT) comparing three different models of pharmacy-delivered PrEP/PEP to a “control” model that represents what pharmacies are currently legally allowed to do in Kenya: screen and refer prospective PrEP and PEP clients to clinic-based services. We hypothesize that enabling pharmacies to initiate and manage clients on PrEP and PEP will lead to higher rates of PrEP initiation and continuation compared to pharmacy referral to clinic-based services.

## METHODS

**Figure 1:**
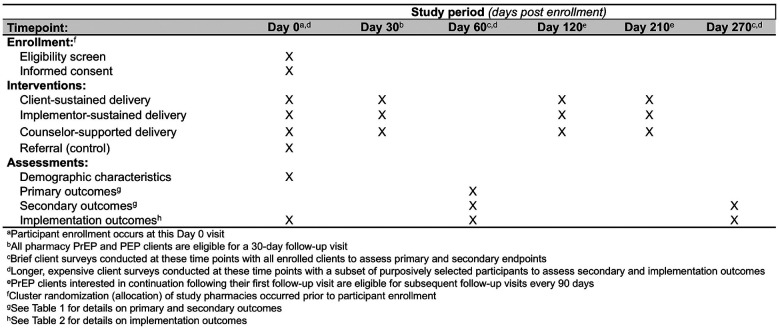
SPIRIT reporting guidelines for interventional trials

We report our cRCT methodology in accordance with the Standard Protocol Items: Recommendations for Interventional Trials reporting guidelines,^17^ Fig. 1**(Additional file 1)**.

### Study design and setting

**Figure 2:**
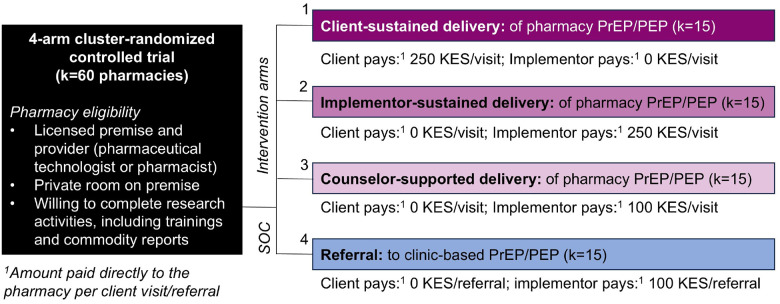
Study design for the Pharm PrEP cRCT

**Figure 3:**
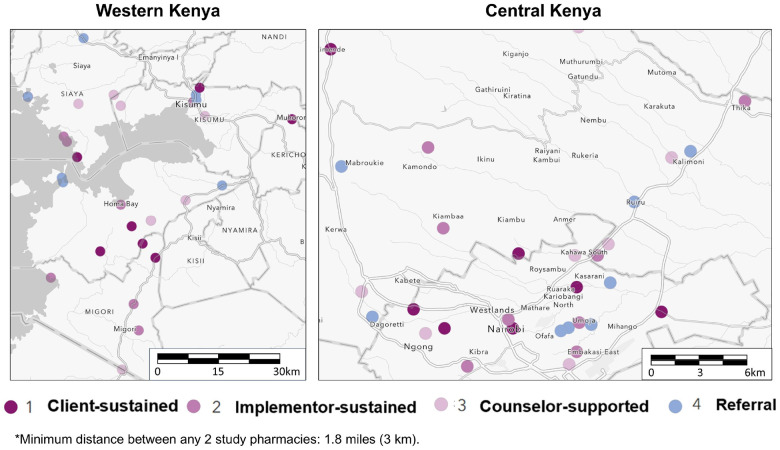
Location of pharmacies randomized to the different study arms

Our four-arm cRCT–the “Pharm PrEP cRCT”–includes 60 community pharmacies or “clusters” (k), equally distributed across four county groups (six counties total) in Central and Western Kenya (ClinicalTrials.gov: NCT05842122), Figs. 2 & 3. In Central Kenya, we are operating in Nairobi County (k = 15) and Kiambu County (k = 15), which include a mix of urban and peri-urban areas surrounded by rural areas and that have a population-level HIV prevalence of ~ 6%.^18^ In Western Kenya, we are operating in Kisumu and Siaya Counties (k = 15, treated as one county group), and in Homa Bay and Migori Counties (k = 15, also treated as one county group); collectively, these two county groups include urban centers surrounded by large rural fishing and farming areas and have a population-level HIV prevalence of ~ 10–17%.^18^

Study pharmacies were stratified by county groups and randomized 1:1:1:1 within groups to either: 1) *client-sustained delivery*, in which clients pay 250 Kenyan Shillings (KES; ~$2 US Dollars [USD]) per pharmacy PrEP/PEP visit; 2) *implementor-sustained delivery*, in which clients pay nothing and implementors pay 250 KES per PrEP/PEP visit, 3) *implementor-sustained + counselor-supported delivery*, in which clients pay nothing, delivery is supported by an HTS counselor stationed at the pharmacy, and implementors pay 100 KES (~$1 USD) per PrEP/PEP visit, or 4) *referral (control)*, in which clients pay nothing and implementors pay 100 KES per PrEP/PEP referral.

In line with Kenya’s interest in public-private partnerships for HIV service delivery,^19^ intervention arm pharmacies are supplied free PrEP/PEP drugs and HIV testing kits from government stock. Pharmacies are compensated for their time spent delivering PrEP/PEP via a fee-for-service model, with the payer and amount varying by arm. We conducted extensive formative research and stakeholder consultation to determine fees that would likely be affordable to a large segment of pharmacies’ clientele, motivating to pharmacy providers, and feasible for a public-sector payer to cover.^20^ In the counselor-supported delivery arm, we opted to provide pharmacies with an HTS counselor, rather than a higher cadre of healthcare provider (e.g., nurses), because HTS counselors are cheaper to employ, in greater supply, and competent in the most time-consuming steps of PrEP/PEP delivery: the HIV risk assessment and HIV testing and counseling.

The Kenyan-based teams implementing this cRCT include two Kenya Medical Research Institute (KEMRI) teams located in Kisumu and Kiambu Counties and a team from Jhpiego, Kenya, located in Nairobi County. These teams–which include site Principal Investigators, study coordinators, technical officers, research assistants, data coordinators, and laboratory technicians–have extensive experience conducting PrEP clinical and implementation research.^2, 15, 21–23^

### Pharmacy eligibility, recruitment, and selection

Pharmacies were eligible for cRCT participation if they were registered with Kenya’s Pharmacy and Poisons Board, were willing to participate in study activities, and had the following: a current practicing license, full-time licensed pharmacist or pharmaceutical technologist, and private room for HIV testing and counseling. We collaborated with county and sub-county health management teams to identify eligible pharmacies located near hotspots for sexual activity–such as universities, bars, and trucking routes. To reduce the risk of contamination between the study arms, we ensured at least three kilometers of distance between selected study pharmacies. In each county group, we identified 20 pharmacies, including five back-ups in the event of study pharmacy attrition.

### Clinic recruitment and engagement

In each county, we worked with the county and sub-county health management teams to identify public, private, and faith-based healthcare clinics located near study pharmacies to which clients could be referred for PrEP, PEP, ART, or other needed services. Additionally, we identified public clinics from which study pharmacies can procure PrEP, PEP, and HIV testing kits; in return, study pharmacies complete monthly dispensing reports that are combined with the clinics’ own consumption data and submitted to the Kenya Ministry of Health (MOH) with resupply requests.

### Pharmacy provider training

All pharmacy providers completed a three-part training: 1) a *self-paced online course*, covering HIV testing, oral PrEP, and PEP; 2) a *two-day interactive in-person course*, to reinforce the online content, review study procedures and logistics, and practice HIV testing (this was implemented by county group); and 3) an *observed practice of HIV testing*, overseen by a Kenya MOH HTS supervisor. In collaboration with the Kenya MOH, we adapted and abbreviated the national PrEP and HIV testing curricula^24, 25^ for pharmacy providers, focusing on the skills and competencies needed for PrEP/PEP delivery that are not part of standard pharmacist or pharmaceutical technologist training programs.

### Randomization

Following completion of the in-person training, one provider per study pharmacy randomly selected an opaque, sealed envelope indicating a study arm assignment or backup pharmacy designation. Study arm assignments were stratified by county group and generated by the study biostatistician, author AM, who was not involved in pharmacy selection, **Additional file 2**. Due to the nature of the intervention, this trial is unblinded. Any time a study pharmacy drops trial participation for any reason (e.g., provider turnover, inability to follow study procedures), that pharmacy is replaced by a randomly selected backup within the same county group.

### Participant eligibility and recruitment

Eligible pharmacy clients are ≥ 16 years old (with those ≤ 17 years meeting the definition of emancipated minors^26^), are interested in PrEP or PEP, meet all criteria on the prescribing checklist (described below), and are willing to consent to study activities. Eligible pharmacy providers, HTS counselors, and remote clinicians are ≥ 18 years old and involved implementation of pharmacy PrEP/PEP delivery or referral. Pharmacy client recruitment is performed across study arms by the trained pharmacy providers, who are encouraged to: display study-supplied posters about PrEP and PEP in their pharmacies (**Additional files 3 & 4**); ask clients seeking sexual and reproductive health products (e.g., condoms, emergency contraception) if they are interested in, or in need of, PrEP or PEP; and share informational brochures with potentially interested clients.

### Ethics

The Scientific Ethics Review Unit of the KEMRI and Institutional Review Board (IRB) of the Fred Hutchinson Cancer Center (Fred Hutch) approved this study protocol. Pharmacy clients complete verbal informed consent after being determined preliminarily eligible for PrEP/PEP and prior to HIV testing or referral; other participants (e.g., pharmacy providers) complete written informed consent prior to engagement in study activities. Study participants receive is 250–500 KES (~$2–4 USD) for completion of each research activity (e.g., surveys), with variations based on data collection tool length. Beyond the fee-for-service amount specified by their study arm assignment, the only additional compensation study pharmacies receive is ~ 5000 KES (~$40 USD) for completion of a pharmacy client volume tracking activity (conducted a maximum of four times).

### Study procedures

**Figure 4:**
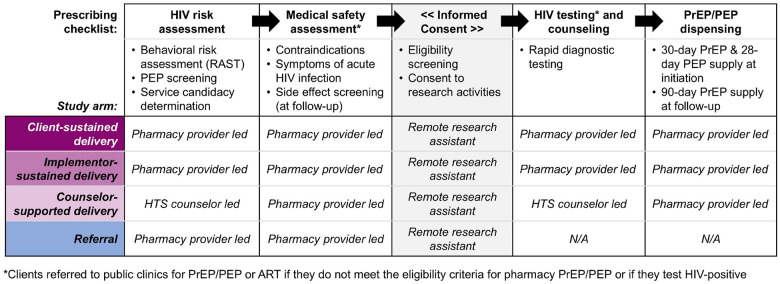
The core components of the prescribing checklist guiding pharmacy PrEP/PEP delivery or referral, by study arm

In all arms, pharmacy providers deliver services using a standardized prescribing checklist, Fig. 4 (see **Additional file 5** for details). In collaboration with Kenyan policymakers,^6^ we developed, refined, and pilot tested^16^ this checklist, which prompts providers to collect basic demographic details about the client then guides them through the following core components of PrEP/PEP delivery: HIV risk assessment, medical safety assessment, HIV testing, and drug dispensing. (Providers in the referral arm use an abbreviated version of the checklist that includes only the client demographics section and HIV risk and medical safety assessments.) An electronic version of the checklist was built into a secure electronic point-of-care sales system (Maisha Meds, Nairobi, Kenya)^27^, with study pharmacies able to complete the checklist on a portable tablet. The system also facilitates weekly payments to pharmacies assigned to study arms in which the implementor pays clients’ PrEP/PEP delivery fees based on submitted checklist data (e.g., number of PrEP/PEP clients served/referred). A remote prescribing clinician is available 24 hours a day, 7 days a week via phone call or WhatsApp to address any pharmacy provider questions or concerns.

After a pharmacy client indicates interest in PrEP/PEP, the pharmacy provider invites them to a private room for eligibility assessment. The checklist assesses HIV risk using with a modified version of Kenya’s HIV Risk Assessment Screening Tool (RAST), which is commonly used at public clinics in Kenya to inform PrEP eligibility.^28^ The modified RAST asks clients whether, in the past six months, they have engaged in nine specific behaviors associated with HIV risk, such as engagement in transactional sex. We modified this tool to additionally ask clients if they perceived themselves to be at risk of HIV acquisition. If the client responded “yes” to any of the modified RAST questions, they were considered to meet the criterion of having HIV risk. To determine whether a client might be eligible for PEP (over PrEP), the checklist additionally asks whether, in the past 72 hours, the client experienced any kind of PEP-qualifying exposure–such as a condom break or shared needles for injection drug use–with someone who may be living with HIV or whose HIV status is unknown. All clients are given the option to self-screen for HIV risk by answering “yes” or “no” to each RAST question on the tablet then allowing the provider to review responses and ask clarifying questions, as needed. PrEP or PEP candidacy is based on clients’ responses to RAST items, and a discussion of their HIV prevention goals and preferences with the pharmacy provider.

Pharmacy providers then screen PrEP/PEP candidates for symptoms of acute HIV infection (e.g., fever, swollen lymph nodes, fatigue); PrEP candidates are additionally screened for a history of liver or kidney disease, diabetes, or hypertension. Across study arms, providers refer clients reporting any of these symptoms/conditions to nearby clinics for PrEP/PEP. Clients who meet the eligibility criteria for pharmacy-based PrEP and PEP are then invited to complete verbal informed consent over the phone with a study research assistant, who confirms a client’s eligibility with the pharmacy provider prior to continuation of PrEP/PEP delivery or referral.

Following consent, providers give clients the option of referral to clinic-based PrEP/PEP or direct pharmacy-based PrEP/PEP depending on their pharmacy’s study arm assignment. In referral arm pharmacies, clients receive information about nearby clinics delivering PrEP and PEP and are given a MOH-style referral form with information about their preferred referral clinic (including its location, hours, and contact information). Clients in referral arm pharmacies interested in HIV testing can purchase self-testing kits, the only permissible form of pharmacy-based HIV testing in Kenya.^25^ In intervention arm pharmacies, provider-administered HIV testing–via rapid diagnostic testing–and PrEP/PEP dispensing are delivered according to national guidelines.^7^ Clients who test HIV-positive are referred to nearby clinics for confirmatory testing and treatment and not eligible for pharmacy PrEP/PEP delivery, while clients who test HIV-negative are eligible to receive either a 30-day supply of daily oral PrEP or 28-day supply of daily oral PEP and scheduled for follow-up 30 days later. To remind clients of follow-up visits, study pharmacies are encouraged to implement whatever systems they utilize for other products requiring follow-up (e.g., insulin). At PrEP follow-up visits, clients again complete the prescribing checklist plus screening for potential PrEP side effects and those eligible are dispensed a 90-day PrEP supply. At PEP follow-up visits, clients receive follow-up HIV testing and are counseled on the potential benefits of PrEP use.

To support pharmacy PrEP/PEP delivery/referral, trained technical officers visit the pharmacies regularly to provide technical assistance (TA) in service provision, commodity acquisition, and report completion. The TA visits vary in frequency based on how long the pharmacy has been participating in the study, rate of participant enrollment, and perceived level of support required.

### Data collection and management

All enrolled pharmacy clients are invited to complete follow-up surveys at 60 and 270 days post-enrollment. We selected 60 days for follow-up to allow clients in the referral arm sufficient time to link to clinic-based PrEP/PEP and clients in all arms time to refill PrEP or transition from PEP to PrEP, if interested. We selected 270 days (~ 9 months) for longer-term follow-up to enable PrEP clients sufficient time to refill PrEP at least three times. The follow-up surveys are brief (~ 10 minutes) and conducted remotely by experienced research assistants. In the surveys, we ask clients to self-report if they initiated PrEP, PEP, or ART (and where) and behaviors and perceptions related to HIV risk acquisition. For clients who initiated PrEP, we ask if they refilled or discontinued PrEP (and when); for clients who initiated PEP, we ask if they transitioned to PrEP or were dispensed PEP again (and when). Using the Wilson et al three-item adherence scale,^29^ we ask clients who initiated PrEP or PEP to self-report their adherence during the first month of use. Participants are considered unreachable after being contacted three times without response.

Other data sources for outcome assessment include pharmacy records; clinic records; longer surveys administered to a subset of clients at baseline, 60 days, and 270 days post-enrollment; dried blood spot (DBS) samples; as well as measurements of pharmacy size (client flow). Pharmacy records are obtained from the electronic prescribing checklist and clinic records are abstracted from referral clinics; both include client-level dispensing information on PrEP and PEP. DBS samples are collected for a subset of clients (n = 200, n = 50/arm) who report PrEP refilling at 60 days; samples are collected within 72 hours of survey completion and will be tested in US-based laboratories for blood levels of tenofovir-diphosphate protective against HIV acquisition.^30^ To estimate the monthly client flow at study pharmacies, we count the number of bags used to dispense products over the course of a month at four time points (i.e., seasons) during cRCT implementation. Pharmacy records are stored in Maisha Meds, while eligibility screening, survey data, clinic record abstraction, and client flow data are recorded in Research Electronic Data Capture (REDCap).^31^

Every week, the study data team merges data from REDCap and Maisha Meds using the participants’ phone or identification numbers. This process supports data quality and prevents duplicate entries. In case of discrepancies, data are checked against study site’s internal records. As a quality control measure, both data entry platforms have error messages that alert the user to out-of-range entries that make the participant ineligible for the study activities.

### Outcomes

Our primary trial outcomes are PrEP initiation and continuation among pharmacy clients determined PrEP eligible by pharmacy providers, Table 1. Both outcomes are self-reported by clients in the first follow-up survey 60 days following enrollment. We categorize PrEP initiation as being dispensed PrEP from any location following enrollment–including at healthcare clinics–and PrEP continuation as any PrEP initiation and refilling (i.e., repeat dispensing). Secondary outcomes among PrEP-eligible clients include PrEP re-initiation (> 7 days after a scheduled follow-up visit), PrEP adherence (assessed via self-report and DBS samples), and behaviors associated with HIV risk at 60 and 270 days; we will also assess primary outcomes secondarily at 270 days from enrollment. Secondary outcomes among PEP-eligible clients include PEP initiation, PEP-to-PrEP transition (i.e., any PrEP dispensing following PEP initiation), repeat PEP use, PEP adherence (self-report), and behaviors associated with HIV risk assessed at 60 and 270 days. Secondary outcomes among all clients include initiation and continuation of any biomedical HIV prevention product (i.e., PrEP or PEP) at 60 and 270 days and recent HIV testing at 60 days.

### Statistical methods

Our primary analysis will be intention-to-treat and use cluster-level data from all enrolled PrEP-eligible clients, including those enrolled in dropped study pharmacies. We will estimate the ratio of PrEP initiation rates (i.e., initiations per pharmacy client served, accounting for pharmacy size) comparing each intervention arm to the referral arm, resulting in three primary comparisons. To do this, we will use Poisson generalized linear models (GLMs) with log links and robust standard errors, adjusted for study arm, pharmacy volume, and county group (to account for stratified randomization). Missing data will be imputed as failures in the primary analysis, and with alternative data sources (i.e., pharmacy and clinic records) or probabilistically (using binomial random variables with the probability equal to the proportion of successes among non-missing observation) in sensitivity analyses.

In secondary analyses, we will compare outcomes in the intervention arms to one another (resulting in three unique comparison groups) and test for differences in intervention effect by urban/rural status and participant subgroups of interest (e.g., women, individuals < 25 years). Additionally, we will compare self-reported outcomes in survey data with those documented in clinic records and compare the proportion of PrEP continuations (among those who initiate PrEP) between each intervention arm and the referral arm. We will use the described Poisson GLM model to analyze secondary rate outcomes and a Poisson generalized estimating equations (GEE) model to analyze secondary proportion outcomes.^32^

### Power

We powered the trial based on our primary PrEP initiation and continuation outcomes. We originally planned to analyze PrEP initiation and continuation through an individual-level analysis comparing the proportion of enrolled participants in each arm but switched in September 2023 to a cluster-level analysis adjusted for pharmacy size (i.e., client flow). This switch was motivated by observed uneven enrollments across the study arms resulting from client interest in the different services offered; if unaccounted for, this would have biased the proportion of PrEP initiations and continuations in each arm. To inform our sample size calculations, we used data on monthly PrEP and PEP initiations from two pilot studies of pharmacy-delivered PrEP and PEP which offered services for a small fee^15^ (like the client-sustained arm) and for free^16^ (like the implementor-sustained arm). Additionally, we used data on PrEP continuation from a large implementation project of clinic-delivered PrEP to inform assumptions in the referral arm.^23^ We hypothesized that the counselor-supported delivery arm would perform best, followed by the implementor-sustained, client-sustained, and referral arms, and adjusted other assumptions appropriately, **Additional file 6**.

To estimate power, we assumed a coefficient of variation of 0.25, typical for cRCTs; a significance level of alpha = 0.05/3, to adjust for multiple comparisons between study arms; and the same average pharmacy size across study arms. We then estimated the number of PrEP initiations needed to detect the smallest hypothesized difference in PrEP initiations between study arms (i.e., the client-initiated vs. referral arms) with 80% power, yielding 1,119 PrEP initiations across arms. This sample provides > 80% power to detect hypothesized differences in PrEP initiation between the other intervention arms compared to the referral, as well as > 80% power to detect hypothesized differences in PEP initiation and continuation and any PrEP or PEP initiation between the intervention arms and referral arm, **Additional file 6**. For our PrEP continuation and any PrEP or PEP continuation outcomes, the sample provides 100% power to detect hypothesized differences between the intervention arms and referral arm with the exception of the client-initiated versus referral arms, in which we only have 23% and 49% power, respectively, to detect our hypothesized differences; while great differences in these outcomes between these arms were not expected, their comparison has other important sustainability implications for pharmacy-delivered PrEP and PEP services.

### Implementation outcomes and determinants

In addition to our primary and secondary trial outcomes, we will assess several implementation outcomes and determinants among clients, providers, HTS counselors, and remote clinicians, Table 2. To assess client perceptions of acceptability and quality of pharmacy PrEP/PEP services received, as well as provider fidelity to PrEP/PEP delivery steps, we will conduct longer surveys with a subset of participants (n = 540) randomly selected at each of the following timepoints: enrollment, 60 days post-enrollment and 270 days post-enrollment. To assess provider and HTS counselor (n = 75) perceptions of acceptability, feasibility, and self-efficacy to deliver PrEP/PEP, we will survey them at enrollment and 60 and 270 days post-enrollment. During routine pharmacy visits, technical officers will document any implementation challenges and actions to address these challenges in a standardized template informed by the Framework for Reporting Adaptations and Modifications to Evidence-based Implementation Strategies (FRAME-IS).^33^ We will conduct microcosting to assess implementation costs in each intervention arm using expense reports, provider surveys about resource use and salaries, pharmacy records, and time-and-motion observations of pharmacy PrEP/PEP visits. Furthermore, we will conduct in-depth interviews (IDIs) with purposively sampled pharmacy clients (n = 148); providers and HTS counselors (n = 55); and remote clinicians (n = 4) to contextualize our quantitative findings (e.g., identify factors that influenced client PrEP/PEP decision-making) and provide insight on implementation, including barriers and facilitators to PrEP/PEP delivery in each study arm, and recommend changes to the delivery models.

### Study oversight

The Fred Hutch team serves as the coordinating center for the trial. This team works collaboratively with Kenyan-based research and implementation teams (described above) to organize virtual weekly meetings, perform data quality checks, monitor cRCT implementation, develop solutions to implementation challenges, ensure protocols and survey tools are up to date, and analyze study data. Fred Hutch team members do not have any direct involvement with study participants.

A Data Safety and Monitoring Board (DSMB)–consisting of two Kenyan- and one US-based HIV researchers, including a biostatistician–is monitoring cRCT implementation. At semiannual DSMB meetings, implementation challenges and solutions are presented along with any reported adverse events (e.g., severe drug side effects, social harms) and primary and secondary trial outcomes (aggregated across study arms in an open session and disaggregated by study arm in a closed session). The DSMB then makes recommendations–related to cRCT implementation, participant safety, data management, quality control, and analysis–which are documented in a report shared with the overseeing IRBs.

### Confidentiality

Study sites use standard operating procedures to ensure participants’ confidentiality before, during, and after trial implementation. Data sets containing participant information are de-identified and records containing identifiable information (e.g., consent forms) are stored and secured separately.

### Dissemination plans

Following the principles of good participatory practice,^34^ we will disseminate study findings to diverse audiences in appropriate formats. To engage participants and other community members, we will present findings at community events and distribute flyers at study pharmacies. To reach Kenyan stakeholders, we will present findings and share policy briefs at national and regional meetings, as well as invited forums with government officials, regulators, researchers, and HIV program implementors. To reach global researchers and policy makers, we will publish peer-reviewed academic manuscripts and present our findings at international HIV conferences.

## DISCUSSION

The three models of pharmacy-delivered PrEP and PEP in the Pharm PrEP cRCT are designed to address different client access barriers (i.e., costs) and provider delivery barriers (i.e., time constraints), while providing important evidence on the potential benefits of direct pharmacy PrEP/PEP delivery compared to pharmacy referral to existing clinic-based PrEP/PEP–which represents the existing standard if governments do not change enabling policies. Additionally, the trial will generate important complementary evidence on barriers and facilitators to implementation of the different delivery models from client and provider perspectives as well as intervention costs. With the inclusion of PEP in addition to PrEP delivery at study pharmacies, the study will also provide insights into clients’ needs and preferences for different biomedical HIV prevention products and help us better understand the role of PEP in HIV prevention programs.

Strengths of this study include the robust cRCT design; measurement of effectiveness and implementation outcomes using mixed methods; delivery of both PrEP and PEP products; three implementation arms testing different pharmacy-delivered PrEP/PEP models; and availability of PrEP/PEP to all pharmacy clients–regardless of gender or age. Other strengths include the involvement of pharmacies across six Kenyan counties and two geographic regions–which will increase the generalizability of our cRCT findings–and our partnership with Kenyan stakeholders to support the study implementation and dissemination of research findings, which will increase the likelihood our findings impact the national scale-up of pharmacy PrEP/PEP in Kenya.

This study also has limitations. For example, study pharmacies and providers were selected based on specific criteria (e.g., availability of a private room) that might not apply to all pharmacies in Kenya; pharmacy-delivered PrEP/PEP services in the intervention arms are supported with government commodities–which lowers implementation costs compared to a non-supported delivery scenario–and pharmacy providers receive TA from study staff to address implementation challenges–which may not be readily available in a scale-up scenario. Additionally, despite the study team’s efforts to ensure appropriate spacing between study pharmacies to avoid contamination between study arms, this was challenging to achieve in areas due to the cost proximity of pharmacies in commercial areas. To mitigate this, we have instructed trained providers to avoid referral to other study pharmacies.

Evidence from this study could guide the Kenya MOH on how public PrEP/PEP commodities could be integrated, scaled, and financed at private pharmacies in Kenya, which could increase the coverage and reach of biomedical HIV prevention products nationally. Additionally, insights on how pharmacy-delivered PrEP/PEP could be implemented in Kenya could impact HIV programming in other countries interested in leveraging private-sector pharmacies for delivery of public HIV commodities–contributing to the goal of ending the global AIDS pandemic.

### Trial status

The first trial participant was enrolled on June 26, 2023; the anticipated last trial participant will be enrolled around July 2025. This trial was registered on ClinicalTrials.gov on April 5, 2023. All relevant ethics committees have approved the trial protocol and relevant modifications; the current protocol was version 2.1 at the time of this publication (February 2025).

## Figures and Tables

**Table 1:** Primary and secondary outcomes of the Pharm PrEP cRCT

Outcomes	Definition	Timing	Data source
***Primary***			
PrEP initiation	Number of participants that initiated (i.e., were dispensed) PrEP at the pharmacy or clinic	60 days	*Brief client surveys;* *pharmacy records;* * *clinic records* *
PrEP continuation	Number of participants that initiated PrEP at the pharmacy or clinic, returned, and refilled PrEP		
**Secondary**			
PrEP initiation	Number of participants that initiated (i.e., were dispensed) PrEP at the pharmacy or clinic	270 days	*Brief client surveys;* *Pharmacy records;* * *Clinic records* *
PrEP continuation	o Number of participants that initiated PrEP at the pharmacy or clinic, returned, and refilled PrEP o Number of participants that initiated PrEP at the pharmacy or clinic and **refilled PrEP at least twice** o Number of participants that initiated PrEP at the pharmacy or clinic and **refilled PrEP more than 7 days after their expected refill date** (i.e., stopping and restarting, also referred to as “PrEP reinitiation”)	270 days	
PEP initiation	Number of participants that initiated (i.e., were dispensed) PEP at the pharmacy or clinic within 3 days of screening	60, 270 days	
PEP-to-PrEP transition	Number of participants that successfully completed PEP and were found eligible for PrEP and initiated PrEP at the pharmacy or clinic	60, 270 days	
PEP/PrEP initiation	Number of participants that initiated (i.e., were dispensed) either PEP or PrEP at the pharmacy or clinic	60, 270 days	
PEP/PrEP continuation	Number of participants that (1) initiated PrEP and refilled PrEP or later were dispensed PEP; or (2) initiated PEP and transitioned to PrEP or later were dispensed PEP again	60, 270 days	
Recurrent PEP use	Number of participants that were dispensed PEP more than one time	60, 270 days	
HIV testing	Number of participants engaging in HIV testing in the 60 days following the pharmacy PrEP/PEP visit	60 days	
PrEP adherence	o Proportion of participants with drug concentrations indicating adherence among those who reported refilling PrEP at 60 days post initiation and were randomly selected for DBS collection and analysis. o Number of participants who initiated PrEP at the pharmacy or clinic and self-reported behaviors indicative of good adherence (using the Wilson et al. scale; 2020, *AIDS)*	60 days	*Extended client survey* *(DBS sample)*
Behaviors associated with HIV risk	Any and specific self-reported behaviors associated with HIV risk acquisition according to Kenya’s Rapid Assessment Screening Tool	60, 270 days	*Brief client survey*
**Exploratory**			
PrEP coverage	Duration of time on PrEP, based on dates of dispensing and the quantity of pills dispensed at each visit using all longitudinal data available at completion of the cRCT	Study duration	*Brief client surveys;* *Pharmacy records;* * *Clinic records* *

* The pharmacy records and client records will be used for imputation, comparison of outcomes between intervention arms, and other secondary/sensitivity analyses

**Table 2:** Implementation outcomes assessed through the Pharm PrEP cRCT

Outcomes	Definition	Timing	Data source
***Client outcomes***			
Acceptability	Using questions that measure different domains of the Theoretical Framework of Acceptability (Sekhon et al., 2017, *BMC Health Serv Res)*, consisting of seven component constructs (e.g., affective attitude, burden, perceived effectiveness)	60, 270 days	*Extensive client surveys* *Client IDIs*
Fidelity	Number and proportion of participants that received different core components of the intervention as specified in the protocol		
Willingness to pay	Amount pharmacy clients are willing to pay for each pharmacy PrEP or PEP visit	60, 270 days	
Quality of care perceptions	Using a modified perceived service quality scale (pSQ-SF6) (Carter et al., 2022, *Res Social Adm Pharm)*, with higher score indicating higher perceived service quality	60, 270 days	
Sustainability	Comparison of study outcomes during the first half and second half of implementation.		*Brief client survey;* *Pharmacy records;* *Clinic records*
**Provider outcomes**			
Acceptability	Using questions that measure different domains of the Theoretical Framework of Acceptability (Sekhon et al., 2017, *BMC Health Serv Res)*, which consists of seven component constructs (e.g., affective attitude, burden, perceived effectiveness)	Baseline, 60, 270 days	*Provider surveys* *Provider IDIs*
Feasibility	Using questions based on the Feasibility of Intervention Measure (FIM) (Weiner et al., 2017, *Implement Sc)*	Baseline, 60, 270 days	
Perceived self-efficacy	Using statements that assess providers’ level of confidence delivering different core components of the intervention	Baseline, 60, 270 days	
Willingness to charge	Amount pharmacy providers are willing to charge for each pharmacy PrEP or PEP visit under different scenarios	Baseline, 60, 270 days	
Adaptations	Using the Framework for Reporting Adaptations and Modifications to Evidence-based Implementation Strategies (FRAME-IS) (Miller et al., 2021, *Implement Sci)*.	Baseline, 60, 270 days	*TA reports;* *Provider surveys*

## Abbreviations

AIDS: Acquired Immunodeficiency Syndrome
ART: Antiretroviral Treatment
cRCT: Cluster-Randomized Controlled Trial
DSD: Differentiated Service Delivery
DSMB: Data Safety and Monitoring Board
DBS: Dried Blood Spot
FRAME-IS: Framework for Reporting Adaptations and Modifications to Evidence-based Implementation Strategies
GEE: Generalized Estimating Equations
GLM: Generalized Linear Models
HIV: Human Immunodeficiency Virus
HTS: HIV Testing Services
IDI: In-Depth Interview
IRB: Institutional Review Board
KEMRI: Kenya Medical Research Institute
KES: Kenya Shillings
MOH: Ministry of Health (Kenya)
PEP: Post Exposure Prophylaxis
PrEP: Pre-Exposure Prophylaxis
RAST: Risk Assessment Screening Tool
REDCap: Research Electronic Data Capture
SPIRIT: Standard Protocol Items: Recommendations for Interventional Trials
TA: Technical Assistance
USD: Unites States Dollar
